# *Agrobacterium*-mediated direct transformation of wheat mature embryos through organogenesis

**DOI:** 10.3389/fpls.2023.1202235

**Published:** 2023-05-31

**Authors:** Xudong Ye, Ashok Shrawat, Lorena Moeller, Rebecca Rode, Anatoly Rivlin, David Kelm, Brian J. Martinell, Edward J. Williams, Anthony Paisley, David R. Duncan, Charles L. Armstrong

**Affiliations:** Plant Biotechnology, Bayer Crop Science, Chesterfield, MO, United States

**Keywords:** wheat, mature embryo, agrobacterium-mediated transformation, centrifugation-assisted *Agrobacterium* inoculation, transgenic plant, multiple shoots, multiple buds, organogenesis

## Abstract

Transgenic plant production in monocotyledonous species has primarily relied on embryogenic callus induction from both immature and mature embryos as the pathway for plant regeneration. We have efficiently regenerated fertile transgenic wheat plants through organogenesis after *Agrobacterium*-mediated direct transformation of mechanically isolated mature embryos from field-grown seed. Centrifugation of the mature embryos in the presence of *Agrobacterium* was found to be essential for efficient T-DNA delivery to the relevant regenerable cells. The inoculated mature embryos formed multiple buds/shoots on high-cytokinin medium, which directly regenerated into transgenic shoots on hormone-free medium containing glyphosate for selection. Rooted transgenic plantlets were obtained within 10-12 weeks after inoculation. Further optimization of this transformation protocol resulted in significant reduction of chimeric plants to below 5%, as indicated by leaf GUS staining and T1 transgene segregation analysis. Direct transformation of wheat mature embryos has substantial advantages over traditional immature embryo-based transformation systems, including long-term storability of the mature dry explants, scalability, and greatly improved flexibility and consistency in transformation experiments.

## Introduction

Common wheat (*Triticum aestivum* L.) is the third largest staple food crop after maize and rice. Sustainable wheat production can significantly contribute to food security for the growing world population (Grote et al., 2021). In addition to conventional breeding, new traits and agronomic performance improvements may be introduced by genetic engineering through foreign gene integration or endogenous gene modification by gene editing tools (Borisjuk et al., 2019; Hayta et al., 2019; Hensel, 2020). Transgenic wheat plants were first obtained from immature embryos (IEs) through somatic embryogenesis by microprojectile bombardment (Vasil et al., 1992) and *Agrobacterium*-mediated transformation (Cheng et al., 1997), respectively, which are still the predominant method for wheat transformation. A method for *in planta* inoculation of *Agrobacterium* suspension into immature seeds in a flowering wheat plant followed by isolation of the inoculated IEs and regeneration of transgenic plants through tissue culture was also developed (Risacher et al., 2009). Wheat IE production usually requires strict growth conditions and timing to obtain optimized explants for transformation. Isolation and inoculation of wheat IEs is an ergonomically tasking skill that limits throughput and scale.

Mature embryos (MEs) from wheat seeds have also been used for transformation through somatic embryogenesis. Transgenic wheat plants were obtained by bombardment of ME-derived embryogenic calli (Patnaik and Khurana, 2003; Miroshnichenko et al., 2020) or by *Agrobacterium*-mediated transformation of precultured MEs on auxin media followed by callus induction and shoot regeneration (Ding et al., 2009; Moghaieb et al., 2010; Aadel et al., 2018; Kumar et al., 2019). *In planta* wheat transformation by direct bombardment of the meristematic tissues of MEs has also been reported. In this method, the coleoptile and the first three leaf primordia were removed from imbibed seeds to expose the shoot apical meristem (SAM), and then the meristem-exposed explants were excised from the seed and arranged for biolistic gene delivery (Hamada et al., 2017).

In recent years, we have developed *Agrobacterium*-mediated direct transformation methods for mature seed embryo explants of soybean, cotton, and maize. Soybean and cotton embryo explants containing the SAM, hypocotyl, and radicle were mechanically isolated, and directly inoculated with *Agrobacterium* containing a binary vector bearing genes of interest (GOI) and the selectable marker gene *aadA* using spectinomycin as a non-lethal selection reagent. A low level of cytokinin [e.g. 1 mg/L thidiazuron (TDZ)] could be included in co-culture medium but was not essential for later shoot formation. Transgenic plants were recovered on hormone-free media with spectinomycin selection through organogenesis (Martinell et al., 2013; Chen et al., 2014). A similar method has been developed for maize transformation, where the maize seed embryo explants contain only the plumule without the radicle after mechanical isolation. Transgenic maize plants are recovered by multiple bud induction on high cytokinin medium followed by shoot regeneration and *de novo* root formation on hormone-free medium with sublethal glyphosate selection (Ye et al., 2022a).

In this paper, we report efficient *Agrobacterium*-mediated direct transformation of mechanically isolated wheat MEs and regeneration of transgenic plants through organogenesis. This novel method has substantial advantages over traditional IE-based transformation systems *via* somatic embryogenesis, because large batches of MEs can be stored for long time (years), which results in cost-savings for donor plant growth, as well as greatly improved flexibility and consistency in transformation experiments.

## Materials and methods

### Mechanical excision of the MEs from wheat seeds

Approximately 5 kg field harvested seeds from the hard red spring elite variety CA905-752 were placed into a perforated metal tray, submerged in sterilization solution with 0.6% v/v sodium hypochlorite and 0.1% v/v Tween 20 for 10 minutes and followed by rinsing three times with sterilized water. After draining the seed tray was transferred to a dehumidifier (Bry-Air Inc. USA, model VFB-3-E-DXA) with settings of the drying temperature at 38°C and the relative humidity at 2% for 48 hours. The explant excision was performed by passing seeds through a modified Grainman rice sheller/dehuller (Grain Machinery MFG. Corp. USA, model #64-115-60-WEC) to recover MEs. The ground wheat particles were purified using a Westrup LA-LS screen cleaner (Westrup A/S, Denmark) which was configured with a US 18x18 stainless steel (SS) woven wire mesh scalp screen, a US 22 x 22 SS woven wire mesh middle screen and a US 35 x 35 SS woven wire mesh bottom screen. The retained material was hand sieved using a US 20 x 20 SS wire mesh screen and US 30 x 30 SS wire mesh screen. All material retained by these two screens makes up the final wheat ME collection. After counting number of embryos per gram of dry material, samples were sealed in mylar packets for immediate use or storage for long term at -20°C. Each batch of wheat MEs were sterilized and plated onto hormone free medium 1083 to establish embryo viability through germination of normal shoots.

### Sterilization and purification of the mechanically isolated wheat MEs

For the proof of concept (POC) transformation experiment, dry wheat MEs were transferred into a 50 mL Falcon tube, covered with 40 mL 70% ethanol, and poured into a deep dish. After two minutes, the ethanol was removed. The MEs were then rinsed five times with 35 mL sterile water containing 0.1% v/v Silwet^®^ L-77 surfactant, soaked for one hour, and replaced with water without Silwet^®^ L-77. At the 2^nd^ or 3^rd^ water replacement, the floating MEs were collected using a small mesh strainer. The process was repeated until all floating MEs were collected. The collected MEs were further soaked in medium 1595 containing 5 mg/L 2,4-D and 0.01% v/v Silwet^®^ L-77 for two hours without stirring before inoculation with *Agrobacterium* suspension.

For the optimized transformation experiments, approximately 10,000 dry MEs were transferred into a sterile glass beaker and covered with 70% v/v ethanol for 4-5 minutes. The MEs were purified by water floatation, soaked in medium 1595 for one to two hours before distributing evenly into two 50 mL Falcon tubes for centrifugation-assisted *Agrobacterium* inoculation.

### Constructs and *Agrobacterium* preparation

Standard cloning procedure was used for vector construction. pMON97367 with the *gus* and *epsps-cp4* expression cassettes is identical to pMON92726 (Ye et al., 2011) except that an additional 333 bp CaMV 35S enhancer sequence resides in the rice actin promoter for stronger *gus* expression. pMON131700 with the *nptII*/*epsps-cp4* dual marker construct was made by inserting *nptII* expression cassette (*P-CaMV35S-nptII-T-nos*) into pMON97367 cut with *SpeI*/*SalI*. pMON264386 contains an *epsps-cp4* cassette driven by the rice tubulin A-3 promoter and a trait gene expression cassette to test in large scale transformation in plant production. pMON138210 is identical to pMON138203 (Ye et al., 2022a) except that the *aadA* coding sequence is codon-optimized for monocotyledonous expression, which was used for localization of the internal GUS expression in **Figure S5**.

All binary vectors are based on *oriV* backbone with spectinomycin selection (Ye et al., 2011) and their T-DNA structures are depicted in **Figure S1**.

The VirG constitutively active *Agrobacterium tumefaciens* strain AB32 (Ye et al., 2016) was used for all wheat ME transformation. Binary vectors were transformed into the AB32 competent cells by electroporation, plated onto LB medium with 50 mg/L spectinomycin and 30 mg/L gentamicin for two days. Single colonies were picked, cultured in LB medium overnight, spun down by centrifugation and suspended in medium 1595 or 3091 (**Table S1**) at OD_600 = _0.5 for inoculation.

### Direct transformation of wheat MEs

After sterilization, 2500 MEs were transferred into a 50 mL Falcon tube, covered with 40-50 mL of *Agrobacterium* suspension and centrifuged at various *g*-force for 30 minutes at 4°C. After centrifugation, the explants were resuspended, poured into a Plantcon^®^ lid, and excess *Agrobacterium* was removed. Approximately 200~300 explants were then spread as a single layer on a sterile 8.2 mm Whatman^®^ filter paper (Cat No. 1001-082) prewetted with 1.25mL of medium 3091 in a deep Petri dish (25 x 85 mm), and co-cultured in a Percival for 3-4 days under light at 23°C and 70% relative humidity.

Filter papers from the co-culture plates were lifted and directly transferred onto 50 mL solid media in deep Petri dishes with or without selection reagents, which were described separately in different experiments during the transformation system development. Cultures were incubated in a Percival under full intensity light (~150 μmol m^-2^ s^-1^) for a 16 hour photoperiod at 25°C for all subculture steps.

For optimized wheat ME transformation, the wheat MEs stored at -20°C up to 2 years were used. The co-cultured explants were transferred onto solid delay medium 3996 (3 mg/L TDZ, 2 mg/L picoloram, 0 μM glyphosate) for two weeks. The explants were then transferred into liquid selection plates containing a filter paper on the top of two felt squares filled with 25 mL medium 3997 (3 mg/L TDZ, 2 mg/L picoloram, 30 μM glyphosate) at a density of 30-50 explants/dish and cultured in the Percival for two weeks under light at 23°C and at least 40% relative humidity. The medium 3997 was removed by aspiration and replaced with 20 mL of hormone-free medium 3995 containing 30 μM glyphosate for selection of two weeks, and further replaced with medium 3995 for another 2 weeks. Excess medium was aspirated after two weeks and replaced with 15 mL of regeneration liquid medium 3995. An overlay of 10 ml of liquid regeneration medium # 3995 was added in each plate after a week. Shoots were harvested from liquid plates and transferred onto solid rooting medium 4237 at ten and twelve weeks post inoculation. All media used in this work are described in **Table S1**.

The T0 plants were defined as the primary transgenic events from the tissue culture and the T1 plants as the progeny plants derived from the self-pollinated seed produced on the T0 plants.

### Histological analysis

The wheat ME explants after co-culture or leaf segments that were collected from plantlets in tissue culture containers or a growth chamber were stained with X-gluc according to the published protocol (Jefferson, 1989). After overnight incubation at 37°C in X-gluc, the leaf segments were de-stained in 70% v/v ethanol and examined for GUS expression.

Wheat ME cross section and toluidine blue staining after embedding in paraffin were performed by Wax-It Histology Service Inc. (http://www.waxitinc.com).

### Molecular analyses of transgenic plants

Genomic DNA was extracted from leaf samples of greenhouse plants as described by Dellaporta et al. (1983). Since the wheat genome is very large (17 Gb), thirty microgram of wheat genomic DNA per lane was loaded for Southern blot using DIG-labeled probes according to manufacturer’s instruction (Roche, Penzberg, Germany).

The transgene copy number and the presence of vector backbone sequence in transgenic plants were determined by Taqman^®^ procedure according to the manufacturer’s instruction (Applied Biosystems, Foster City, CA). The primers and minor groove binding (MGB) Taqman^®^ probes were designed and validated by the manufacturer. The RK2 oriV probes for backbone detection were described previously (Ye et al., 2008). The primers used for DIG-labeling and the Taqman^®^ transgene copy number determination are summarized in **Table S2**.

### Statistical analysis

Paired t-test was used to calculate statistical significance of the TF between treatments. Data were presented as mean ± standard errors. Chi-square test was used for transgene segregation analysis of T1 seeds.

## Results

### Mechanically excised wheat MEs are viable for plant regeneration

**Figure 1:**
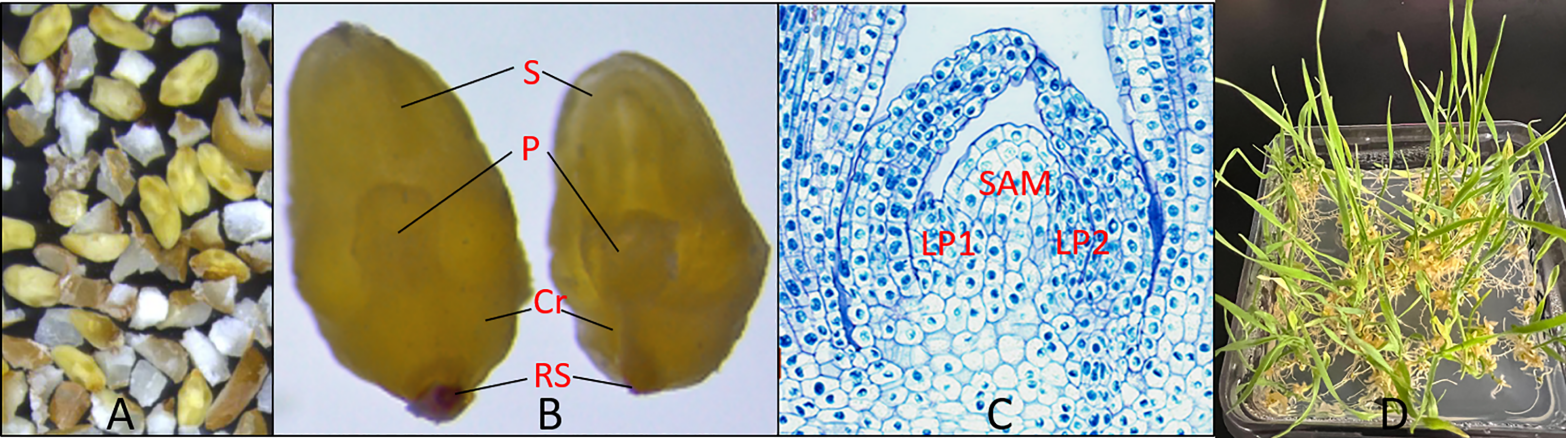
Machine-excised wheat MEs. **(A)** Wheat embryos (yellow) and starchy endosperm debris (white) after mechanical excision from dry seeds. **(B)** Enlarged view of two intact wheat MEs. S: Mature scutellum, P: plumule, Cr: coleorhiza, RS: remains of suspensor (Lersten, 1987). **(C)** Cross-section of a plumule of wheat ME with toluidine blue staining. The SAM region is labeled. LP1 and LP2 are leaf primordia 1 and 2. **(D)** Plantlets regenerating from wheat MEs after three weeks on a hormone-free medium.

Wheat MEs were mechanically excised from field grown seeds by a modified duhuller and purified through a series of wire mesh screens. The process for mechanical isolation of wheat mature embryos is described in the section of Materials and Methods. The majority of the mechanically isolated MEs were intact, including a large scutellum, and the complete embryo axis with coleoptile and primary root (**Figure 1A**, yellow, and enlarged in **Figure 1B**; Lersten, 1987). The wheat MEs (**Figure 1A**, yellow) are often mixed with starchy endosperm debris (**Figure 1A**, white). The SAM is surrounded by leaf primordia and enclosed in the coleoptile (**Figure 1C**). The SAM can be directly regenerated into normal plants on hormone-free medium 1083 (**Table S1** **;** **Figure 1D**).

### Centrifugation-assisted *Agrobacterium* inoculation improved T-DNA delivery into wheat MEs

Wheat MEs were poorly transformed by *Agrobacterium* inoculation after extensive testing with conventional sonication, vacuum and/or surfactant treatments. We discovered that inoculation of wheat MEs by centrifugation in the presence of *Agrobacterium* suspension drastically improved GUS transient expression.

**Figure 2:**
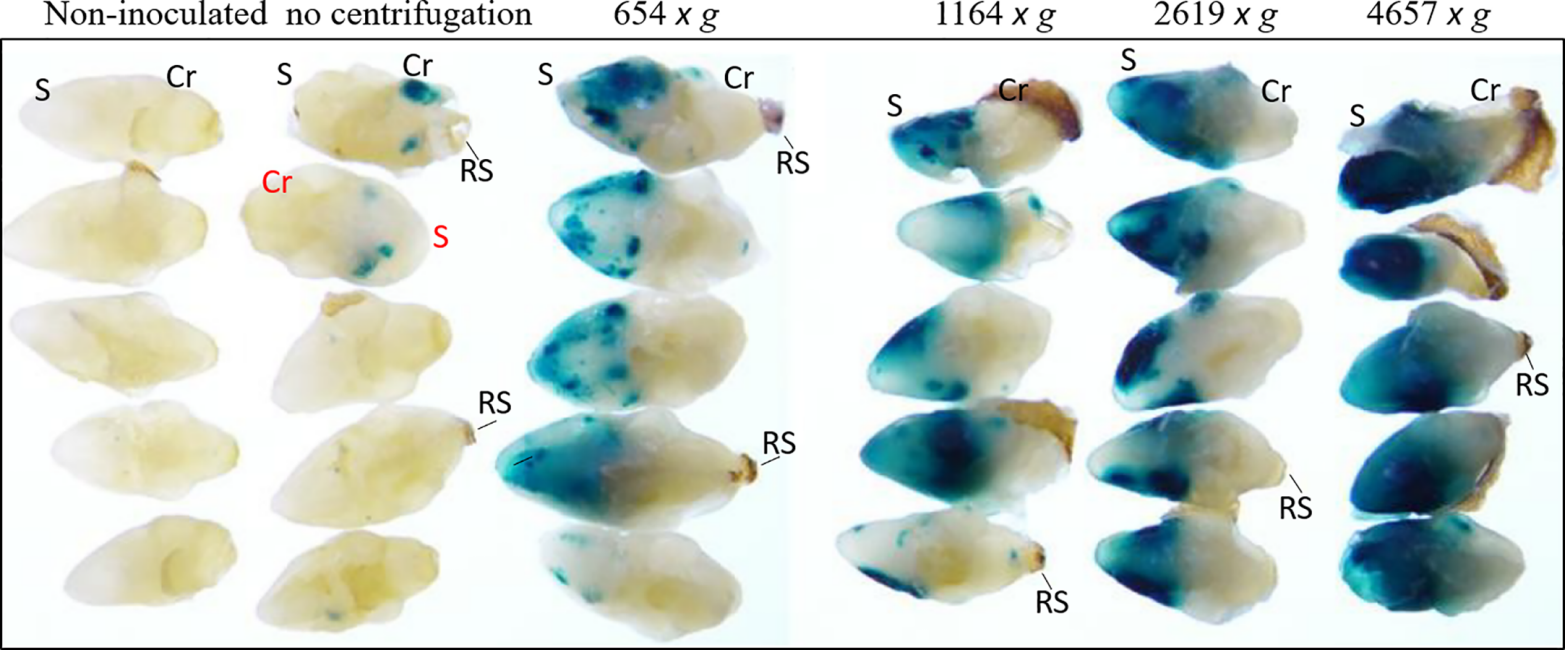
Centrifugation-assisted *Agrobacterium* inoculation drastically increased transient GUS expression in wheat ME surfaces. *Agrobacterium* AB32/pMON97367 was used in this experiment. In all treatments, MEs were centrifuged for 30 min at 4 °C. The GUS staining was performed after co-culture. The centrifugation force used is labeled above the picture. The relative centrifugal force (RCF) is marked as *x g* (*g*-force). Mature scutellum (S), coleorhiza (Cr) and the remains of suspensor (RS) positions are marked.

*Agrobacterium* AB32/pMON97367 culture was suspended in inoculation medium 3091 (**Table S1**) containing 5 mg/L 2,4-D and 0.002% v/v Silwet^®^ L-77 and used for inoculation of wheat MEs with various centrifugation forces. As shown in **Figure 2**, the control without centrifugation showed poor GUS transient expression, which was drastically improved by applying gravity forces (*g*-force) up to 4657 *x g*. GUS staining was observed primarily on scutellum surfaces around plumules leading to necrosis in subsequent glyphosate selection. However, GUS staining was also localized internally in meristem regions, leaf bases and coleoptiles of co-cultured wheat MEs after fixation and paraffin emedding (**Figure S5**). Many of our early experiments used 291 or 654 *x g* for convenience (e.g. 1000 rpm or 1500 rpm setting), while 1400 *x g* was used for our optimized transformation protocol, which formed loose pellets to be resuspened easily.

### Proof of concept for direct transformation of wheat MEs with the *nptII* and *epsps-cp4* dual selectable markers

The success in direct transformation of maize seed embryo explants (Ye et al., 2022a) inspired us to test direct transformation of the mechanically isolated wheat MEs using similar media. The initial attempt for multiple shoot induction from wheat MEs on the maize multiple shoot induction medium (CMSI-18 with 1 mg/L 2,4-D and 10 mg/L BAP; **Table S1**) failed since most wheat MEs turned brown even without selection. The MEs on CMSI-2 containing 3 mg/L TDZ and 2 mg/L picloram remained green and formed some multiple shoots after *Agrobacterium* inoculation (**Figure S2**). Therefore, we decided to use CMSI-2 for direct transformation of wheat MEs.

**Figure 3:**
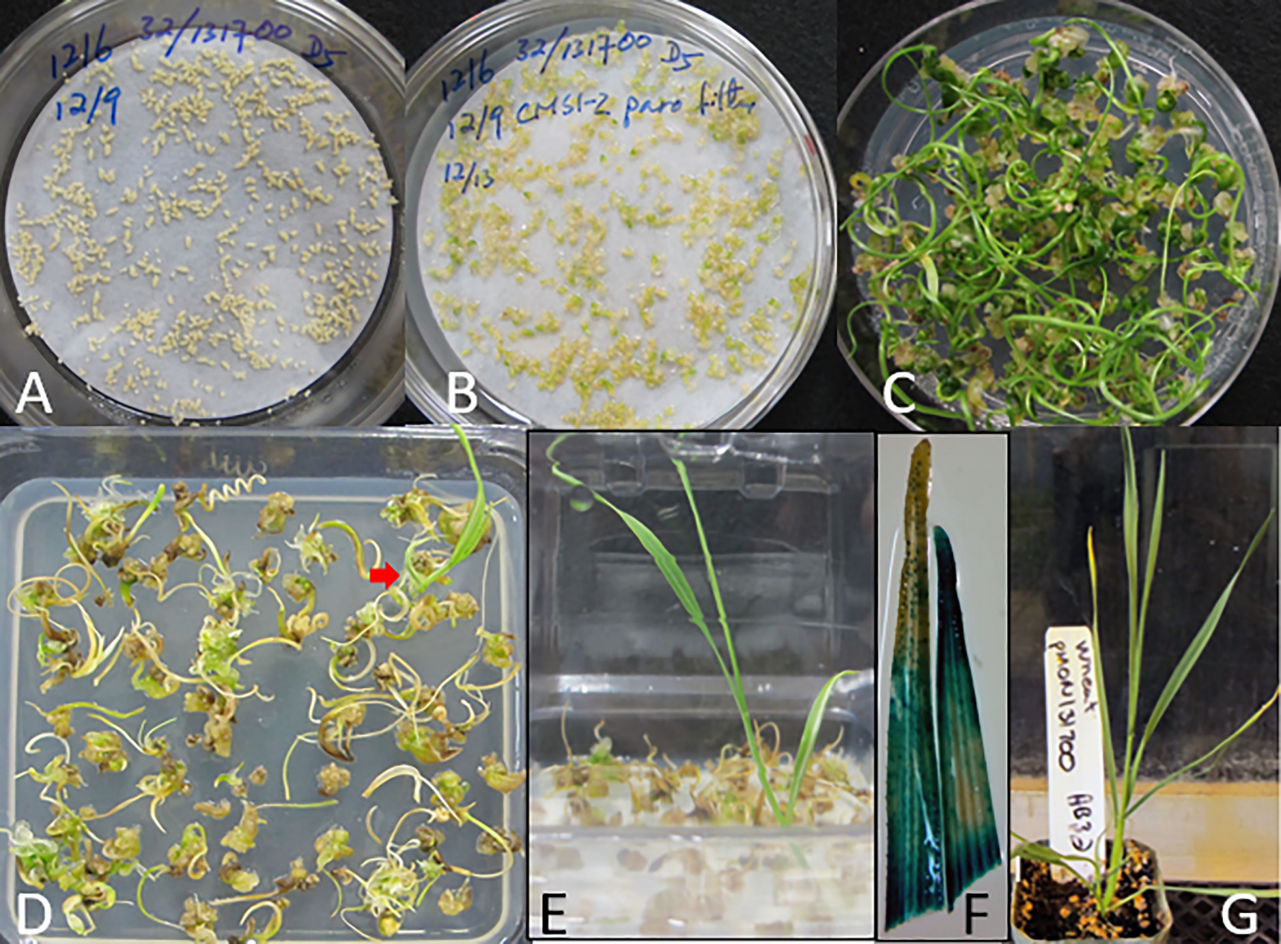
Transgenic plant from direct transformation of wheat MEs with *nptII* and *epsps-cp4* dual selection. **(A)** After co-culture, filter paper with the inoculated MEs is transferred onto CMSI-2 with 50 mg/L paromomycin. **(B)** The explants swell and begin to green after 4 to 5 days on the paromomycin medium. **(C)** Extensive green shoot formation after five weeks on CMSI-2 with paromomycin selection. **(D)** After an additional three weeks on hormone-free medium (CMSI-63) containing 50 μM glyphosate, a green shoot with a root (indicated by an arrow) was recovered. **(E)** A well developed shoot with roots after four weeks on a selection medium containing 50 μM glyphosate. **(F)** GUS staining in the second and third leaves from the plant shown in **(E)**. **(G)** The first transgenic wheat event in soil.

Approximately 2650 wheat MEs were purified by floatation and inoculated with *Agrobacterium* AB32/pMON131700 (containing *nptII/epsps-cp4* dual selectable markers) in medium 1595 and 0.002% Silwet^®^ L-77, followed by centrifugation for 30 minutes at 291 *x g* at 4 °C. The pellet of wheat MEs and *Agrobacterium* cell mixture was resuspended by shaking and the *Agrobacerium* suspension was completely removed by aspiration. Approximately 300 wheat MEs were plated onto a 8.2 cm round filter paper wetted with 1.5 mL medium 1595 containing 5 mg/L 2,4-D in a deep Petri dish (25 x 85 mm) (**Figure 3A**). After three days of co-culture at 23 °C, the filter paper with the inoculated wheat MEs was transferred onto CMSI-2 medium containing 50 mg/L paromomycin (**Figure 3A**). Surprisingly, paromomycin did not inhibit the primary shoot elongation from wheat MEs. Instead, the mature wheat scutella were swollen, while the embryonic leaf primordia and SAMs directly germinated into shoots after five weeks (**Figure 3C**). For selection of potential transgenic shoots, we terminated the paromomycin selection and transferred the clumps with elongating shoots onto the hormone-free medium CMSI-63 (**Table S1**) containing 50 μM glyphosate. After three weeks on glyphosate selection, most green shoots from the paromomycin selection turned brown, but a few green shoots remained healthy and developed a root (**Figures 3D, E**). The first three leaves from one of these plants were analyzed for GUS expression, and the staining indicated that they could be transgenic events. (**Figure 3F**). A total of five events from this initial experiment were transplanted into soil approximately nine weeks after inoculation, and grown to maturity in the greenhouse for seed production.

**Figure 4:**
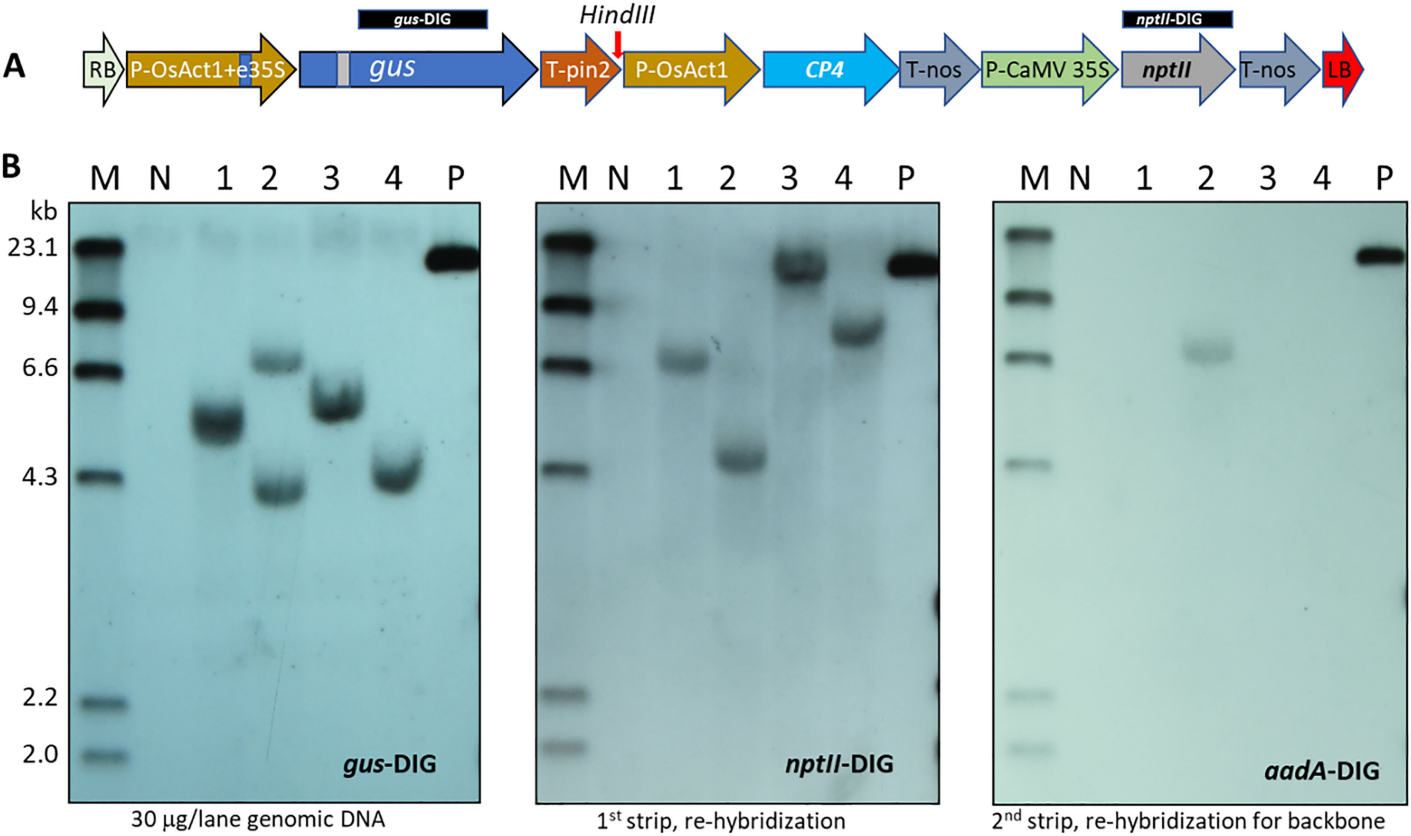
Transgene confirmation of wheat ME-derived events transformed with *Agrobacterium* AB32/pMON131700. **(A)** T-DNA structure of pMON131700 for wheat transformation. Vertical arrow indicates *HindIII* unique site. The DIG labeled 1014 bp *gus* and 809 bp *nptII* probes are marked above the T-DNA. The *aadA* probe is 691 bp, which is in the vector backbone, outside of the T-DNA borders. **(B)** Southern blot detection for transgene insertion with the *gus*, *nptII* and *aadA* probes, respectively. The same membrane was hybridized with the DIG-labeled probes sequentially after being stripped according to the manufacturer’s instructions. M: DIG-labeled molecular size marker of *HindIII* digested Λ DNA; N: non-transgenic wheat CA905-752 genomic DNA as a negative control; 1-4: plant samples from four transgenic wheat plants, 30 μg/lane genomic DNA is loaded; P: 20 pg pMON131700 plasmid DNA as a positive control. The DIG labeled probes are indicated in each hybridization.

To confirm stable integration of the T-DNA into the nuclear genome, four of the five initial proof-of-concept (POC) events derived from the *nptII*/*epsps-cp4* dual selection were analyzed by Southern blot. The fifth event was not evaluated by Southern due to severe chimeric GUS expression detected in leaves (**Figure S4**). As revealed by the *gus* and *nptII* probes in **Figure 4**, transgenic event #2 showed two copies of the T-DNA integration and presence of vector backbone sequence as detected by the *aadA* probe. The other 3 transgenic events appeared to have single copy and backbone-free insertions of the T-DNA.

### Wheat ME transformation using immediate glyphosate selection after inoculation

**Figure 5:**
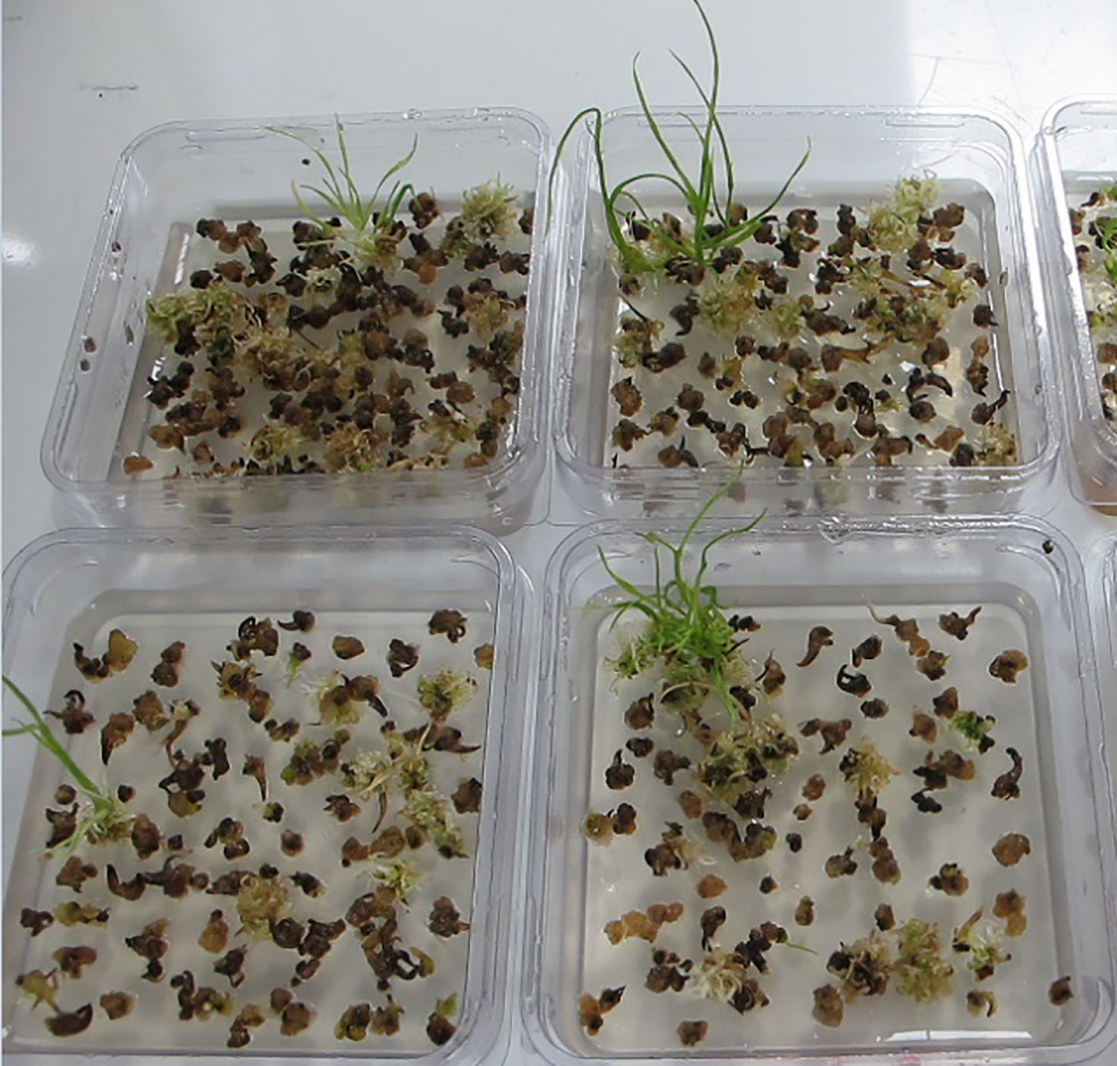
Glyphosate-resistant wheat plantlets derived from MEs using glyphosate direct selection. Explants displayed severe necrosis in about five weeks with immediate glyphosate selection after co-culture with *Agrobacterium.*.

In the POC experiment, glyphosate selection was critical to obtain the transgenic plants, whereas the paromomycin selection did not show significant inhibition during first five weeks of selection on the bud induction medium (**Figure 3C**). To test if transgenic plants could be obtained with direct glyphosate selection, approximately 1700 MEs were inoculated with *Agrobacterium* AB32/pMON97367 suspension containing 5 mg/L 2,4-D and 0.01% v/v Silwet^®^ L-77 and subjected to centrifugation at 2600 x *g* for 15 minutes at 4 °C. The inoculated MEs were co-cultured on a single filter paper in a deep Petri dish under light at 23 °C for four days. Following co-culture, the filter papers containing MEs were directly transferred onto CMSI-2 medium containing 10 μM glyphosate for two weeks, followed by subculture onto CMSI-2 with 30 μM glyphosate for an additional three weeks. The swelling and slightly discolored greening clumps were further transferred onto hormone-free CMSI-63 containing 30 μM glyphosate for shoot elongation and root formation. Glyphosate-resistant shoots with well developed root system were observed within three weeks following transfer of green clumps onto the CMSI-63 selection medium. Although, the majority of wheat explants showed necrosis (**Figure 5**), 11 events were recovered on CMSI-63 selection medium. These events were confirmed by GUS staining of leaf segments and transplanted into soil with a transformation frequency (TF = #transgenic events/#ME explants x 100) of 0.65%.

### Removal of 2,4-D and Silwet^®^ L-77 from the co-culture medium drastically increases wheat ME transformation frequency

Since immediate glyphosate selection resulted in explant necrosis on regeneration medium (**Figure 5**), we tested a delay selection step to improve wheat ME explant health for supporting shoot formation. In the optimized glyphosate selection protocol, a two-week delay (no selection) step on solid medium 3996 (**Table S1**) was included, which was followed by glyphosate selection. The subculture efficiency was improved by liquid selection which enabled aspiration of old medium and replacement of fresh medium without changing containers.

**Figure 6:**
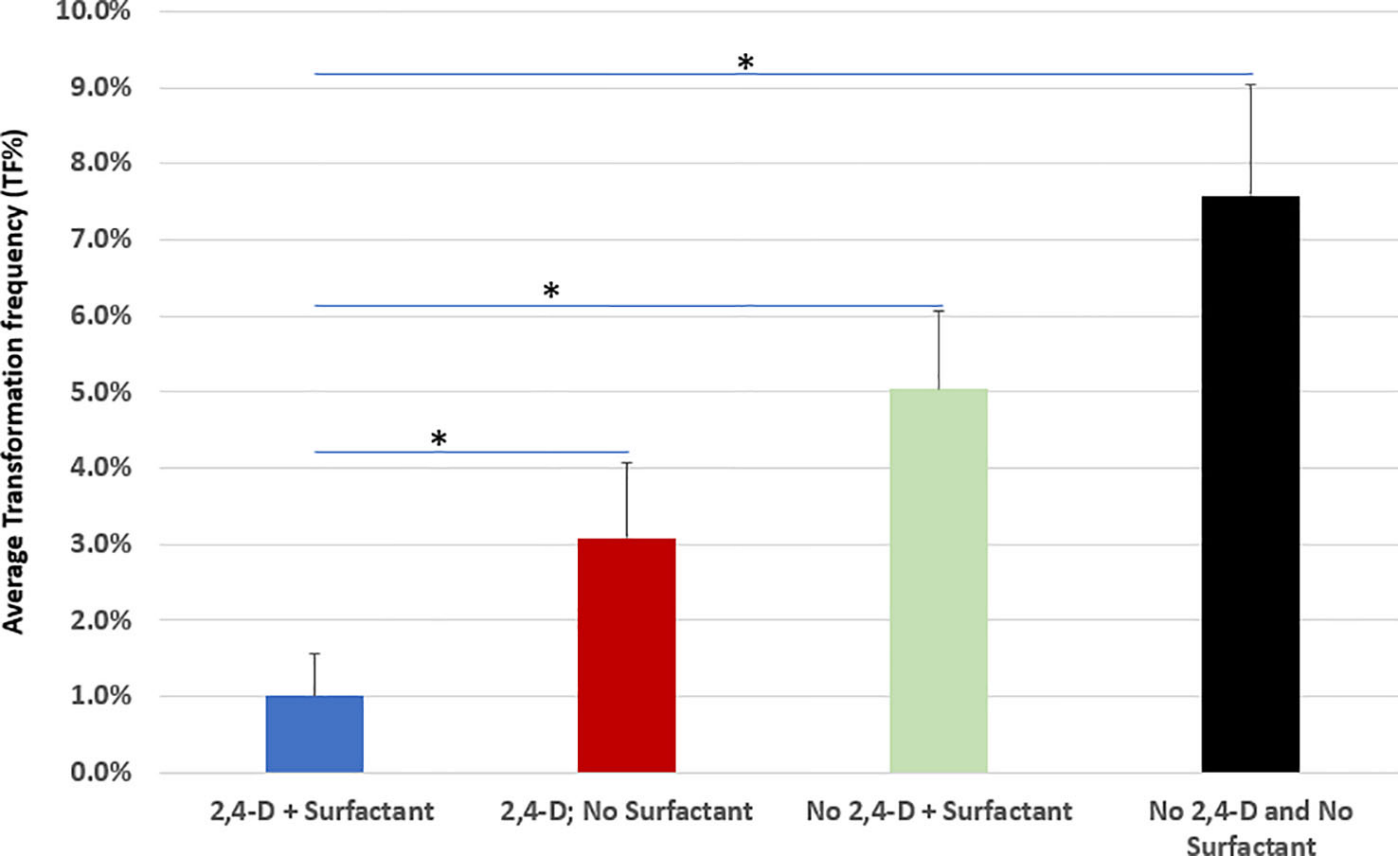
Removal of 2,4-D auxin and Silwet^®^ L-77 surfactant drastically improves transformation frequency. AB32/pMON264386 (**Figure S1**) was used for all transformation. Bars represent averages of TF% of biological replicates for each treatment. *A paired t-test was used to analyze TF improvement significance (*p*<0.05). For each treatment, >10,000 explants were used per treatment and each treatment had at least three biological reps.

In comparison to the pilot transformation protocol (**Figure 6**, the 1^st^ bar) where 5 mg/L 2,4-D and 0.005% v/v Silwet^®^ L-77 surfectant were added to the co-culture medium for wheat ME transformation, the removal of either Silwet^®^ L-77 (**Figure 6**, the 2^nd^ bar) or 2,4-D (**Figure 6**, the 3^rd^ bar) from the co-culture medium improved TF significantly (*p*<0.05), while removal of both 2,4-D and Silwet^®^ L-77 (**Figure 6**, the 4^th^ bar) from the co-culture medium further increased TF more than 7-fold compared to the control (**Figure 6**). Transformed MEs without 2,4-D and Silwet^®^ L-77 in co-culture medium became greener, had elongated green leaves, and induced healthy green multiple buds/shoots during 14 days of delay selection period (**Figure S3**). We observed that on average 25% of T0 events were single copy and backbone-free events with a range of 18~32% across experiment replications.

**Figure 7:**
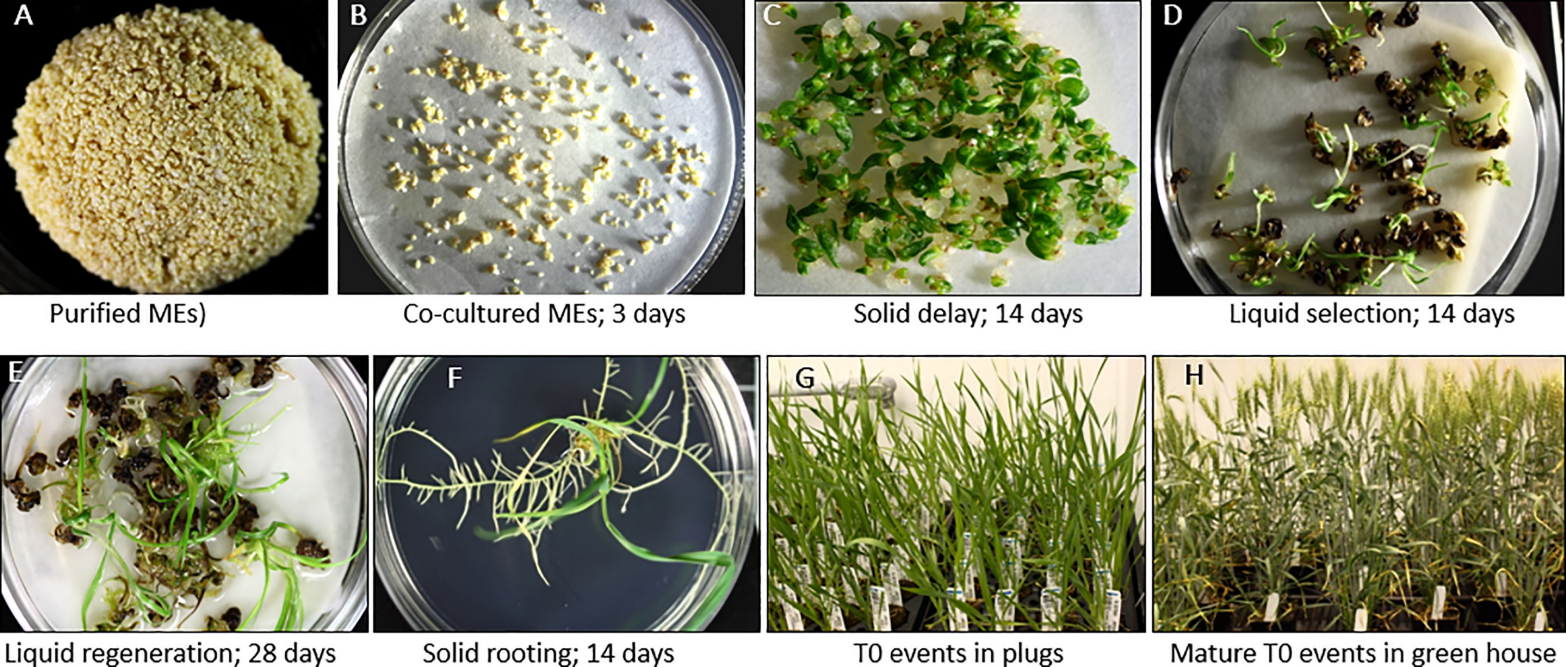
*Agrobacterium*-mediated direct transformation of wheat mature embryos. **(A)** Purified wheat MEs ready for transformation. **(B)** Co-cultured MEs. **(C)** Solid delay medium # 3996 (The co-culture filter paper containing wheat mature embryos was directly transferred onto the top of the solid delay medium). **(D)** Selection of multiple buds/shoots on liquid selection medium, **(E)** Regeneration on liquid regeneration medium. **(F)** Rooting on solid rooting medium. **(G)** T0 transgenic events in plugs. **(H)** Mature T0 transgenic events in the green house.

The detailed wheat ME direct transformation protocol is depicted in **Figure 7**. The media and subculture details are described in **Table 1**.

**Table 1:** Workflow for *Agrobacterium*-mediated direct transformation of wheat MEs.

Steps	Details of the steps	Duration/Culture conditions
*Agrobacterium* preparation	100 ml of LB + 200 µl of glycerol stock (AB32 strain carrying a desired plasmid) overnight - the overnight OD_660_ should be between 0.8-1.0 before centrifugation. Final *Agrobacterium* inoculum is suspended in medium 3091 and final OD_660_ is set to 0.25.	
Sterilization	Pour entire Mylar bag containing 20,000 MEs into a 1 liter sterile bottle and add 1 liter 70% ethanol. Sterilize MEs by slowly agitating the MEs for 4-5 mins. Collect the MEs by floating with 8 liters of water.	
Inoculation	Approximately 2500 MEs are combined with 40-45 ml of *Agrobacterium* inoculum in a 50 ml centrifuge tube. The MEs are then centrifuged at 1400 x g at 4 °C for 30 min.	
Co-culture: Medium 3091	1.25 ml of medium 3091 is added to co-culture plate containing 1 sterile filter paper. Approximately 156 MEs are placed onto the wet filter in a plate and spread out evenly with a sterile spatula.	3-4 days in the dark at 23 °C/70% humidity-put the plates (in stack)
Solid delay: Medium 3996	Filter papers from co-culture plate are lifted and directly transferred to solid delay medium 3996 plates	2 weeks
Liquid selection: Medium 3997	2 felts and 1 filter paper plate with a hole - 25 ml of selection medium 3997 was added in each plate. Note: 1 solid delay plate was divided into 5 selection plates	2 weeks
Regeneration: Medium 3995	Selection medium is aspirated completely after 2 weeks and 20 ml of liquid regeneration medium 3995 was added in each plate	2 weeks
Aspiration of regeneration medium	Excess medium is aspirated and replaced with 15 ml of fresh regeneration liquid medium 3995	1 weeks
Overlay	An overlay of 10 ml of fresh liquid regeneration medium 3995 is added in each plate after a week	1 weeks
1^st^ Rooting: Medium 4237	Shoots are harvested from liquid plates and transferred to solid rooting medium 4237 at 8 weeks post inoculation	2 weeks
2^nd^ Rooting: Medium 4237	Shoots are harvested from liquid plates and transferred to solid rooting medium 4237 at 10 weeks post inoculation	2 weeks

After co-culture, all subcultures are incubated in a Percival under full intensity light (~150 μmol m^-2^ s^-1^), 16-hour photoperiod at 25°C.

### Investigation of T0 chimerism and T1 transgene transmission of the transgenic plants derived from direct transformation of wheat MEs

To investigate the chimera frequency, leaf segments from multiple tillers of each POC event were collected for GUS staining. Events #1-3 showed uniform GUS expression in leaf edges of all tillers, indicating the regeneration of non-chimeric transgenic shoots. In contrast, light and severe chimerism was observed in events # 4 and 5, respectively, as evidenced by uneven GUS staining (**Figure S4**).

The T1 transgene transmission of the four T0 events from the POC transformation with paromomycin and glyphosate dual selections (**Figure 3**) was also analyzed by GUS staining of leaf segments. Events # 2 and #3 displayed the 3:1 normal Mendelian segregation ratio as expected for a single functional insert. Since the Southern data for event # 2 indicated the presence of two T-DNA inserts for this event, it is likely that these inserts are closely linked in the genome. Alternatively, the inserts could be genetically unlinked but the *gus* gene is either absent or expressing at levels below the limit of detection by the X-gluc assay for one of the inserts. Events #1 and #4 had a significantly reduced frequency of GUS positive T1 plants, indicating that both T0 events were likely chimeric (**Table 2**).

**Table 2:** T1 transgene transmission of the four POC events from the direct transformation of wheat MEs.

T0 event #	Southern copy #	T1 seedlings	GUS^+^	GUS^-^	GUS^+^: GUS^-^
1	1	40	7	33	chimera
2	2	106	76	30	2.53: 1
3	1	88	63	25	2.52: 1
4	1	54	1	53	chimera

GUS, leaf staining with X-gluc solution; GUS^+^, GUS staining positive; GUS^-^, GUS staining negative. Event #2 is a two-copy event by Southern blot detection, the other three events are single copy.

We further investigated T1 transgene transmission of forty randomly selected single-copy and backbone-free T0 events produced using the optimized transformation protocol (no 2,4-D and Silwet^®^ L-77 in the co-culture medium; two week delay selection, followed by glyphosate selection). Sixty seeds per event were planted and sampled. The *epsps-cp4* Taqman^®^ probe was used to detect hemi- or homozygosity of T1 plants. We observed that 97.5% of the events (39 out of 40) showed normal Mendelian segregation and 95% of the events (38 out of 40) had six or more homozygous transgenic T1 progeny (**Supplementary, data sheet 2**), indicating low chimerism in the T0 events and a high frequency of germline transmission when using the optimized transformation protocol.

## Discussion

We have developed an efficient transformation method for the production of transgenic wheat plants through organogenesis by *Agrobacterium*-mediated direct transformation of MEs. The ME-based direct transformation system provides high flexibility for wheat transformation as the MEs are amenable to long term storage and can be used for transformation at any time without the laborious maintenance of donor plants for IE isolation or induction embryogenic calli for gene delivery.

In comparison to maize seed embryo explants, which only contain the plumule and lack the radicle and scutellum (Ye et al., 2022a), the majority of the mechanically isolated wheat MEs are intact. Like maize and many other monocotyledonous species, the SAMs of wheat MEs are enclosed within multiple layers of leaf primordia and the coleoptile, which need to be removed for physical access when using biolistic DNA delivery technology (Hamada et al., 2017). It was demonstrated previously that inoculation of *Agrobacterium* by centrifugation improved the TF in rice and maize IE transformation (Hiei et al., 2006) and greatly enhanced the transformability using maize seed excised explants (Ye et al., 2022a). In this study we also found that inoculation of MEs with *Agrobacterium* by centrifugation was critical for the development of an efficient direct transformation system of wheat MEs. Transient GUS expression as a visible marker is a very helpful tool to evaluate the T-DNA delivery efficiency because the intensity of GUS expression on explants is thought to be an indicator for cells with T-DNA delivery. The duration of centrifugation and *g*-forces appeared to influence transient GUS expression on scutellum surfaces as shown in **Figure 2**. Since the entire scutellum turned to necrosis under glyphosate selection, the transgenic events were likely derived from SAM or meristematic cells inside enclosed coleoptile (**Figure S5**). We used 1400 *x g* for 30 min in our optimized protocol because it formed loose pellets to be resuspened easily and gave the best TF in large scale transgenic plant production. Further optimization may be required based on the stable transformation data with a wide range of centrifugation treatments.

A medium with 3 mg/L TDZ and 2 mg/L picloram was previously used to induce multiple shoots/buds from excised wheat meristematic shoot segments of 5-7 day germinating wheat MEs. The shoot elongation from these multiple shoots/buds was carried out on medium containing 1 mg/L TDZ and 0.1 mg/L picloram, while the rooting step required 0.5 mg/L indole-3-butyric acid (Sharma et al., 2005). We used similar media for direct induction of multiple shoots/buds from the intact wheat MEs after *Agrobacterium* inoculation. Transgenic shoot elongation and rooting did not require additional plant growth regulators.

The *nptII* selectable marker was previously used for *Agrobacterium*-mediated transformation of wheat IEs in combination with G418 (Cheng et al., 1997), or paromomycin as a selection reagent (Xue et al., 2004), both of which belong to the aminoglycoside class of antibiotics. It was shown that paromomycin at 15 mg/L gave about 50% regeneration from wheat calli and at 25 mg/L concentration killed all wheat seedlings (Jhinjer et al., 2017). Surprisingly, paromomycin selection of wheat MEs for five weeks did not inhibit wheat ME germination (**Figure 3C**). The different responses between MEs and IEs to paromomycin may indicate that the sensitivity of the explants is different, and a higher concentration of paromomycin may be required to inhibit ME germination.

In meristem explant-based transformation systems, a living non-transgenic tissue may be required to support transformed cell growth during the early stages of transformation. In these protocols, the mature embryo explants do not undergo callus formation. Shoot formation relies on organogenesis from meristematic cells in or near the SAM region, and nutritional supply to the meristematic cells must depend on supporting tissues from the original embryo structure. Optimization of the selectable marker and selection conditions is necessary to maintain or regenerate supporting tissues. For example, in soybean meristem explant transformation, a sublethal concentration of glyphosate (25 μM) was used for the elongation of transgenic shoots, which can adversely damage the original hypocotyl and radicle, such that the elongated shoots must be cut for rooting (Ye et al., 2008). The non-lethal selectable marker *aadA* with spectinomycin selection in both soybean and cotton meristem explant transformation confers much advantage over glyphosate for direct rooting in soil plugs without an extra rooting step because the original hypocotyl and radicle are still viable after selection and can resume normal rooting in the absence of spectinomycin (Martinell et al., 2013; Chen et al., 2014). The same holds true for maize seed embryo explant transformation where the immediate glyphosate selection resulted in poor transformation since most explants showed severe necrosis four weeks after culture initiation. We found that delaying selection for one or two weeks greatly improved explant viability and efficient multiple bud formation allowing subsequent shoot elongation under sublethal glyphosate selection (Ye et al., 2022a). Similarly, we also observed in wheat ME transformation that immediate selection at a low concentration of glyphosate resulted in necrotic clump formation in five weeks after culture initiation (**Figure 4**). Delaying the start of selection by two weeks was found to be important to improve the viability of wheat ME explants for shoot formation.

In our initial attempts for wheat ME transformation, we included 5 mg/L 2,4-D during co-culture to promote cell division (Garcia et al., 2019) and Silwet^®^ L-77 (Cheng et al., 1997) to improve *Agrobacterium* access to internal meristematic cells while we had not figured out yet how to regenerate transgenic shoots through embryogenesis or organogenesis. The same medium was also used for co-culture of maize seed embryo explant in the early stage of method development (Ye et al., 2022a). It was noteworthy that removal of 2,4-D and the surfactant from the co-culture medium greatly increased TF in the ME direct transformation system through organogenesis. The presence of 2,4-D during co-culture may have directed the transformed meristematic cells toward an embryogenic pathway instead of organogenesis, which resulted in low frequency of transgenic shoot regeneration (**Figure S3**). In *Agrobacterium*-mediated transformation of wheat IEs, the presence of surfactant during inoculation improved the delivery of T-DNA to the target tissue (Cheng et al., 1997). Similarly, the use of Silwet L-77^®^ in combination with high sucrose and glucose, tobacco leaf extract, and acetosyringone improved the efficiency of genetic transformation of foxtail millet (Sood et al., 2020). However, in our study, we did not see a beneficial effect of surfactant on transformation and, in fact, removal of the surfactant Silwet^®^ L-77 dramatically improved TF. A negative effect of Silwet^®^ L-77 and 2,4-D in the co-culture medium was also observed for maize seed embryo explant direct transformation (Ye et al., 2022a).

Regeneration of chimeric plants are always a concern in meristem explant-based transformation through organogenesis because they skew the T0 plant transgene copy number determination and reduce T1 transmission rate. In our ME transformation method, we observed that over 90% of soybean T0 events and over 80% of cotton T0 events from meristem explant transformation had normal T1 transmission rates (Ye et al., 2022b). This may be attributed to efficient spectinomycin translocation to meristems (Pyke et al., 2000) resulting in stringent selection. In our recent maize seed embryo explant direct transformation, we were able to reduce chimeric plant regeneration frequency from ~ 80% to ~50% by implementing an extended multiple bud induction step to sort out transformed buds through cell proliferation under selection (Ye et al., 2022a). In wheat ME direct transformation, the frequency of chimeric plants was very low (less than 5%; **Supporting Information 2**) after the introduction of a two week delay selection step. In the wheat ME POC experiment (**Figure 3**) with five week paromomycin selection followed by glyphosate selection, we observed that two out of the four analyzed transgenic plants showed severe T1 transmission reduction even though the Southern blot of T0 leaf DNA showed comparable hybridization signals among the four events (**Figure 4**), indicating a uniform or high percentage of transgene integration in leaf samples. GUS staining of leaf segments from the two T0 events with severe T1 transmission reduction revealed even leaf edge staining from all five tillers in event #1 and only a few chimeric streaks from two tillers in event #4 (**Figure S4**). The reduced T1 transmission in the two POC events may result from poor paromomycin selection for germline cells in the first five weeks, suggesting that the leaf tissues and the germline cells have different progenitor cells, and that the germline cells may form between two and five weeks in the ME direct transformation since the no selection delay step of two weeks supports over 95% T1 transmission.

The method we developed for wheat direct ME transformation may be applicable to many graminaceous species that share similar embryo structure to wheat. Since currently many graminaceous species require IEs from greenhouse grown plants or lengthy induction of embryogenic calli from mature seeds for transformation (Singh and Prasad, 2016), the direct transformation of MEs through organogenesis can provide great flexibility in transgenic plant production.

## Data availability statement

The original contributions presented in the study are included in the article/**Supplementary Material**. Further inquiries can be directed to the corresponding author.

## Author contributions

XY: wheat ME direct transformation POC, constructs, Southern blot, manuscript drafting. AS and RR: critical TF improvement by removing 2,4-D and surfactant. LM: T-DNA delivery improvement, histological observation, T1 chimerism investigation. EW: centrifugation inoculation. RR and DD: transformation system optimization. AR, DK and BM: wheat ME excision. CA: critically revised the manuscript. All authors contributed to the article and approved the submitted version.
