# Supplementary material for: Agrobacterium-mediated direct transformation of wheat mature embryos through organogenesis
Source: Front Plant Sci. 2023 May 31;14:1202235. doi: 10.3389/fpls.2023.1202235 (PMC10264787; doi:10.3389/fpls.2023.1202235)
Supplement: Supplementary file 1 [file DataSheet_1.docx]

**Table S1: Media used for direct transformation of wheat mature embryos**

| **Medium name** | **Base media** | **Cytokinin** | **Auxin** | **Comments** |
| --- | --- | --- | --- | --- |
| 1595 | B5 basal salts  + B5 vitamins | 0 | 0 | **Inoculation medium**: 3.5 g/L dextrose + 3.9 g/L MES + 1 g/L potassium nitrate, pH 5.4, autoclaved |
| 3901 | 1/10 MS medium  + MS vitamins | 0 | 0 | **Inoculation medium**: 40 g/L maltose + 0.1 g/L casein hydrolysate + 0.5 g/L glutamine + 0.75 g/L magnesium chloride + 1.95 g/L MES + 100 mg/L ascorbic acid, pH 5.8, filter sterilized |
| 1083 | MS medium | 0 | 0 | **Shoot regeneration medium**: 10 g/L glucose + 20 g/L maltose + 0.15 g/L + 0.15 g/L asparagine monohydrate + 0.1 g/L myo-inositol |
| CMSI-2 | MS medium | 3 mg/L TDZ | 2 mg/L picloram | **Multiple bud induction**: 30 g/L maltose + 0.5 g/L L-proline + 1 g/L casein hydrolysate + 0.2 g/L myo-inositol + 1 mg/L thiamine hydrochloride + 1.25 mg/L cupric sulphate + 200 mg/L carbenicillin + 100 mg/L cefotaxime. pH 5.8, solidified with 3.5 g/L Agarose |
| CMSI-18 | MS basal salts  + B5 vitamins | 10 mg/L BAP | 1 mg/L 2,4-D | **Multiple bud induction**: 30 g/L sucrose + 0.69 g/L L-proline + 1 g/L casein hydrolysate + 2 mg/L glycine + 1 g/L MES + 400 mg/L carbenicillin + 200 mg/L cefotaxime + 100 mg/L Timentin, pH 5.8, solidified with 3.5 g/L Agarose |
| CMSI-63 | MS medium | 0 | 0 | **Regeneration and selection medium:** 30 g/L sucrose + 0.69 g/L L-proline + 1 g/L MES + 50 μM glyphosate + 400 mg/L carbenicillin + 200 mg/L cefotaxime + 100 mg/L Timentin, pH 5.8, solidified with 3.5 g/L Agarose |
| 3996 | 0.78 g/L MS salts without nitrogen  + MS vitamins | 3 mg/L TDZ | 2 mg/L picloram | **Solid multiple bud induction without selection (delay medium)**: 60 g/L maltose + 1.64 g/L potassium sulphate + 4.95 g/L ammonium nitrate + 0.5 g/L glutamine + 1 g/L NZ amine-A + 1.95 g/L MES + 0.75 g/L magnesium chloride hexahydrate + 1.25 mg/L cupric sulphate + 200 mg/L carbenicillin + 100 mg/L cefotaxime, pH 5.8, solidified with 3.5 g/L Agarose |
| 3997 | 0.78 g/L MS salts without nitrogen  + MS vitamins | 3 mg/L TDZ | 2 mg/L picloram | **Liquid multiple bud induction with selection**: 30 g/L maltose + 1.64 g/L potassium sulphate + 4.95 g/L ammonium nitrate + 0.5 g/L glutamine + 1 g/L NZ amine-A + 0.75 g/L magnesium chloride + 1.95 g/L MES + 1.25 mg/L cupric sulphate + 200 mg/L carbenicillin + 100 mg/L cefotaxime + 30 μM glyphosate; pH 5.8, filter sterilized |
| 3995 | MS medium  + MS vitamins | 0 | 0 | **Liquid regeneration medium with selection**: 30 g/L sucrose + 0.69 g/L L-proline + 1 g/L MES + 400 mg/L carbenicillin + 200 mg/L cefotaxime + 100 mg/L Timentin + 30 μM glyphosate, pH 5.8, filter sterilized |
| 4237 | MS medium  + MS vitamins | 0 | 0 | **Rooting medium with selection:** 40 g/L maltose + 0.5 mg/L cupric sulphate 0.5 mg/L + 1.95 g/L MES + ascorbic acid 100 mg/L + 30 μM glyphosate + 400 mg/L carbenicillin + 500 mg/L cefotaxime.  *Medium solidified with 3 g/L Gelzan* |

**Note:** **MS medium** consists of MS basal salts and vitamins; **TDZ**: thidiazuron; **BAP**: 6-benzylaminopurine; **picloram**: 4-amino-3.5,6-trichloropicolinic acid; **2,4-D**: 2,4-Dichlorophenoxyacetic acid; **MES**: 2-(N-morpholino)ethanesulfonic acid

**Additional references:**

Murashige, T. and Skoog, F. (1962) A revised medium for rapid growth and bioassays with tobacco tissue cultures. *Physiol. Plant.* **15**, 473–497. <https://doi.org/10.1111/j.1399-3054.1962.tb08052.x>

Gamborg, O.L., Miller, R.A. and Ojima, K. (1968) Nutrient requirements of suspension cultures of soybean root cells. *Exp Cell Res*. **50**, 151–8. <https://doi.org/10.1016/0014-4827(68)90403-5>

**Table S2: Primers used for PCR synthesis of DIG-labeled probes for Southern blots and transgene copy number determination**

| **Detection of gene** | **Forward primer** | **Reverse primer** | **TaqMan^®^ probe** |
| --- | --- | --- | --- |
| *gusA* DIG probe | 5’ ACGATATCACCGTGGTGACGC 3’ | 5’ CACTCCACATGTCGGTGTACA 3’ | - |
| *nptII* DIG probe | 5’ TCGCATGATTGAACAAGATGGA 3’ | 5’ AGAGTCCCGCTCAGAAGAACTC 3’ | - |
| *aadA* DIG probe | 5’ GAAGTATCGACTCAACTATCAGAGG 3’ | 5’ AGCGATCTTCTTGTCCAAGATAAGC 3’ | - |
| *epsps-cp4* Taqman | 5’ TACGATTTCGACAGCACCTTCA 3’ | 5′ GTCACCGTCTTCCGATTTCAC 3′ | 5’ ACGCCTCGCTCACAAAGCGCC 3’ |
| Internal control *(ALMT1*) | 5’ AATGACTGTGCCGTCTCCAGT 3’ | 5’ ACAGAGCCGTGTTCTCTGCA 3’ | 5’ CGTGAAAGCAGCGGAAAGCCTCAGA 3’ |

**Note:** Wheat *ALMT1* gene GenBank accession # DQ072262. The [TaqMan^®^](https://www.ulab360.com/files/prod/manuals/201603/13/554048001.pdf) probes were labeled on the 5’-end with the 6-carboxy-fluorescein (FAM) reporter dye or other dyes for multiplex PCR and with the 6-carboxytetramethylrhodamine (TAMRA) quencher dye attached to the 3’-end by ABI (Applied Biosystems Co., Ltd., Foster City, CA).

**Additional references:**

Bubner, B. and Baldwin, I.T. (2004) Use of real-time PCR for determining copy number and zygosity in transgenic plants. *Plant Cell Rep.* **23**, 263–271. <https://doi.org/10.1007/s00299-004-0859-y>

Raman, H., Zhang, K., Cakir, M., Appels, R., Garvin, D.F., Maron, L.G., Kochian, L.V., Moroni, J.S., Raman, R., Imtiaz, M., Drake-Brockman, F., Waters, I., Martin, P., Sasaki, T., Yamamoto, Y., Matsumoto, H., Hebb, DM., Delhaize, E. and Ryan, P.R. (2005) Molecular characterization and mapping of *ALMT1*, the aluminium-tolerance gene of bread wheat (*Triticum* *aestivum* L.). *Genome*. **48**, 781–91. <https://doi.org/10.1139/g05-054>


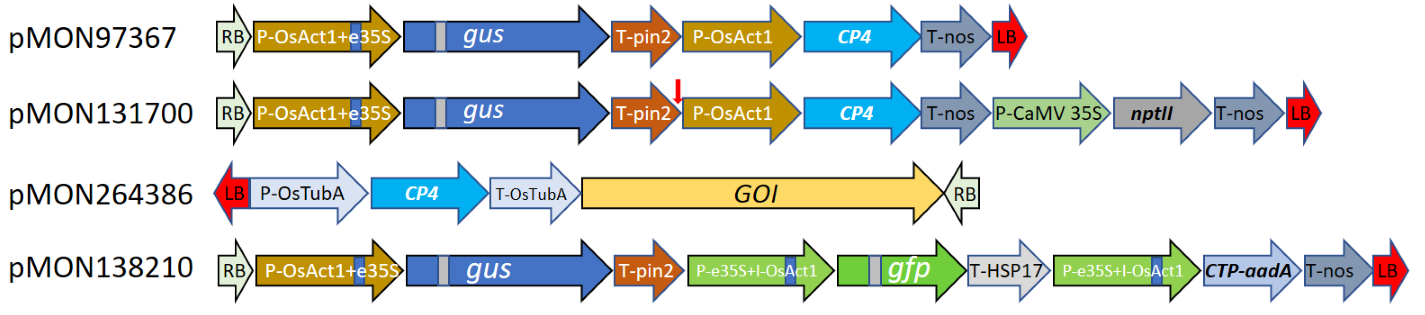


**Figure S1: Constructs used for wheat mature embryo direct transformation. *P-OsAct1+e35S***: rice actin 1 promoter with CaMV enhancer sequence; ***gus****: gusA gene with intron (*Vancanneyt et al., 1990); ***T-pin2****:* Potato proteinase inhibitor II terminator (GenBank accession X04118)*;* ***CP4****: epsps-cp4 gene from Agrobacterium* CP4 strain encoding for 5-enolpyruvulshikimate-3-phosphate synthase; ***T-nos****: Agrobacterium nos* transcription terminator (Depicker et al. 1982); ***P-eCaMV35S***: enhanced CaMV 35S promoter; ***T-HSP17***: wheat heat shock protein terminator (GenBank accession X13431); ***gfp****:* green fluorescent protein gene (Pang et al., 1996); ***nptII***: neomycin phosphotransferase gene; ***CTP-aadA***: chloroplast transit peptide fused to a codon optimized *aadA* marker gene; ***P-OsTubA***: rice alpha tubulin-3 (*TubA-3*) promoter (GenBank accession MH931401.1); ***T-OsTubA***: rice alpha tubulin-3 (*TubA-3*) 3’ UTR (GenBank accession MH931402.1); ***GOI***: a trait gene as a gene of interest for testing transformation.

**Additional references:**

Depicker, A., Stachel, S., Dhaese, P., Zambryski, P. and Goodman, H.M. (1982) Nopaline synthase: transcript

mapping and DNA sequence. *J Mol Appl Genet.* **1**, 561–73.

Pang, S.Z., DeBoer, D.L., Wan, Y., Ye, G., Layton, J.G., Neher, M.K., Armstrong, C.L., Fry, J.E., Hinchee, M.A.

and Fromm, M.E. (1996) An improved green fluorescent protein gene as a vital marker in plants. *Plant Physiol.* **112**, 893–900.

Vancanneyt, G., Schmidt, R., O’Connor-Sanchez, A., Willmitzer, L. and Rocha-Sosa, M. (1990) Construction of an

intron-containing marker gene: splicing of the intron in transgenic plants and its use in monitoring early events in *Agrobacterium*-mediated plant transformation. *Mol Genet Genom*. **220**, 245–2503.

**
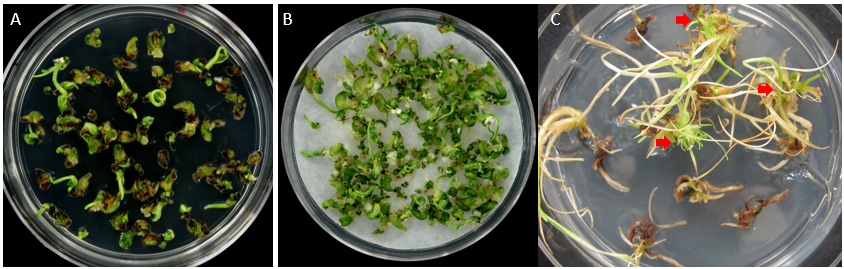
**

**Figure S2: Multiple shoot formation from wheat MEs on CMSI-2.** (**A**) Wheat MEs were placed on CMSI-18 (1 mg/L 2,4-D and 10 mg/L BAP) solid medium for three weeks. Explant necrosis was observed on CMSI-18 solid medium. (**B**) Wheat MEs were placed on a filter over two layers of felts in CMSI-2 (2 mg/L picloram, 3 mg/L TDZ) liquid medium for 3 weeks. **(C)** Multiple shoot formation (red arrows) on CMSI-2 solid medium after inoculation with *Agrobacterium* suspension in an early transformation attempt.


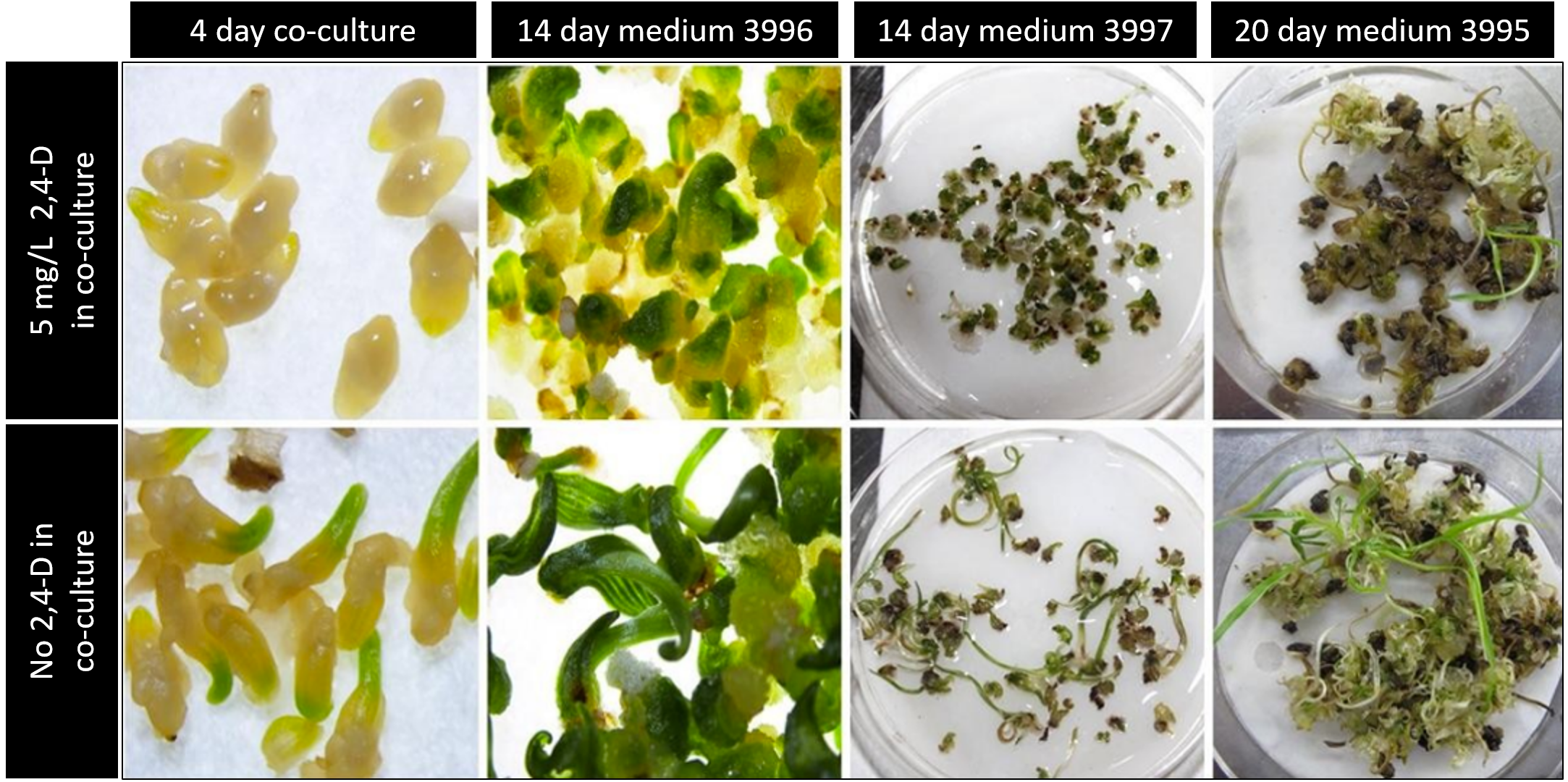


**Figure S3. Removal of 2,4-D from inoculation and co-culture steps results in improved explant health.** The subculture durations on different media are labeled on top. Upper panel with 5 mg/L 2,4-D in co-culture. The lower panel without 2,4-D in the co-culture medium.


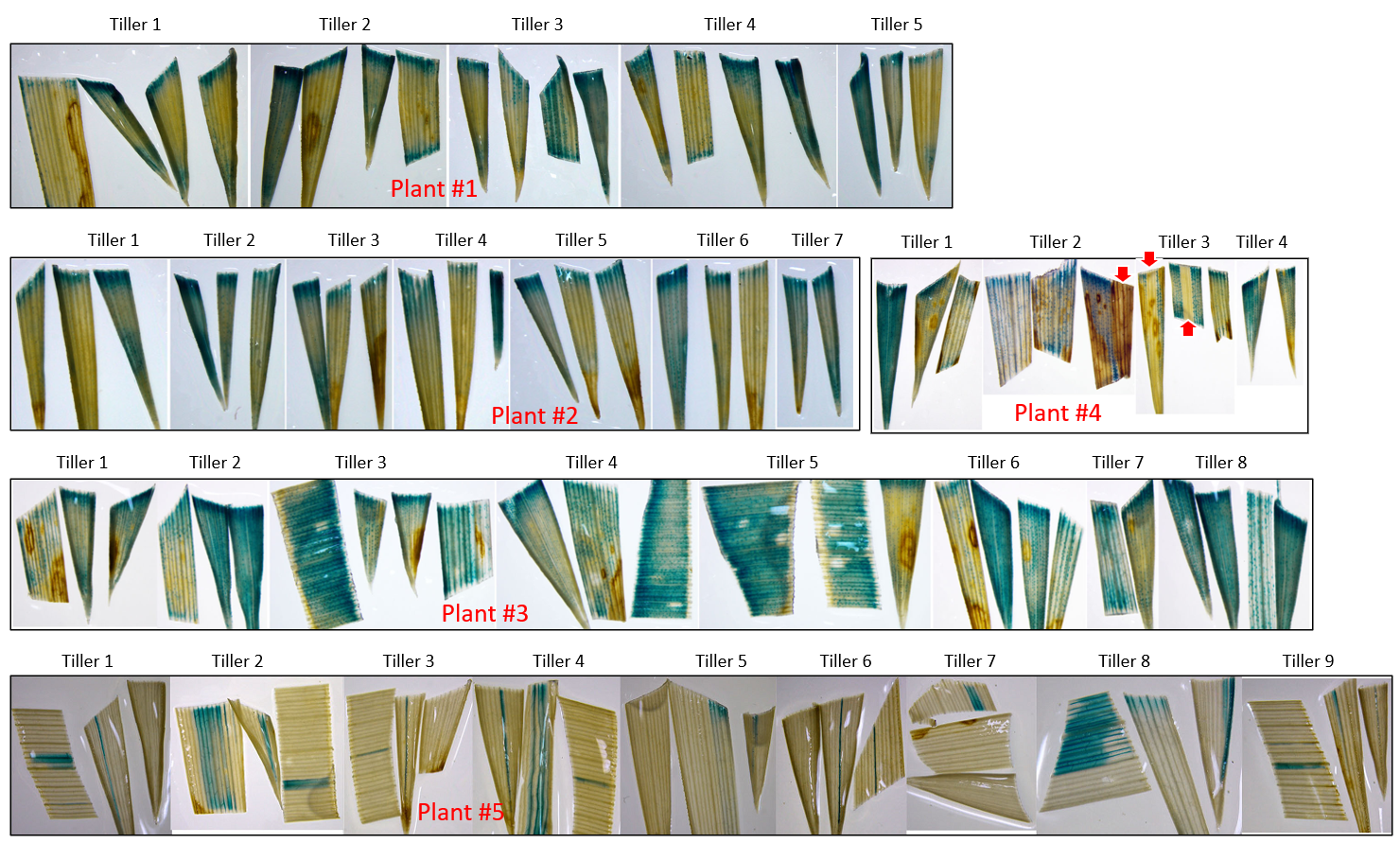


**Figure S4**: **GUS staining of multiple tillers of the 5 wheat transgenic plants from the initial POC experiment**. Leaf segments from 2-4 leaves of each tiller were collected for GUS staining. Arrows in the plant #4 indicate non-transgenic stripes. Plant #5 was severely chimeric and not analyzed by Southern blot. Plant #2 and #3 showed normal T1 transmission, while plant #1 and #4 showed reduced T1 transmission (Table 1) although Southern blot (Fig. 4) revealed equivalent detection signals in leaf samples.


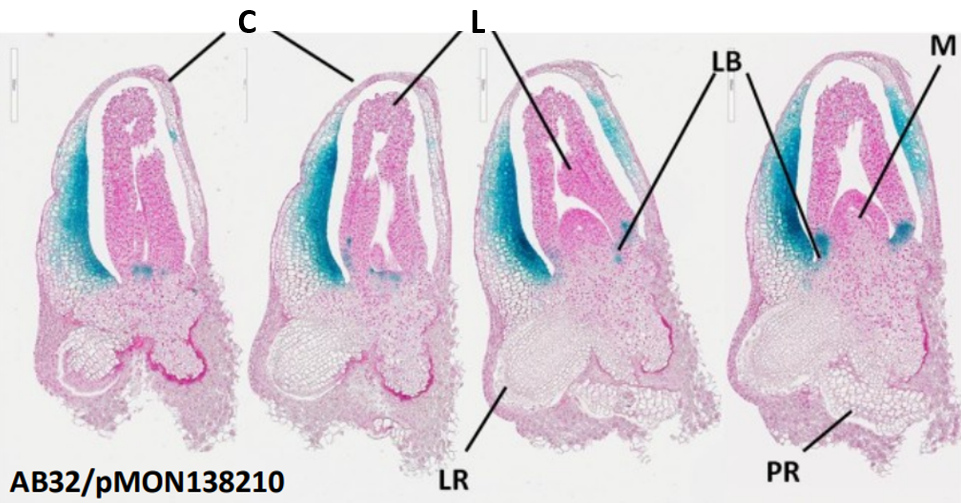


**Figure S5: Localization of GUS transient expression inside wheat MEs inoculated with *Agrobacterium***. C: coleoptile; L: leaf; LB; leaf base; M: meristem; LR: lateral roots; PR: primary root.

**Procedure**: Wheat MEs were inoculated with AB32/pMON138210 at 0.5 OD660 (centrifugation 30 min at 2916 *x* *g*). Explants were co-cultured on one filter paper with 1.25 mL of inoculation medium 3091 for 3 days at 23 ^o^C. Ten positively expressing GFP explants were selected for X-Gluc staining overnight and fixed in FAA solution (45% ethanol, 5% acetic acid, 3.7% formaldehyde. For 100mL, mix 45 mL ethanol, 5 mL acetic acid, 10 mL formalin, 40 mL sterile distilled water). Explants were vacuum infiltrated in cold FAA on ice for 15 min. Fixative was replaced and fixation continued overnight at 4°C in a rotator shaker. Fixative was replaced with 70% ethanol at room temperature for 1h. Ethanol was replaced with fresh ethanol, and explants were stored at 4°C until ready to ship. The fixed co-cultured wheat MEs were embedded in paraffin, sectioned and counter stained with ruthenium red by Wax-It Histology Service Inc.
